# A simple predictive model for estimating relative e-cigarette toxic carbonyl levels

**DOI:** 10.1371/journal.pone.0238172

**Published:** 2020-08-26

**Authors:** Shawna Vreeke, Xijing Zhu, Robert M. Strongin

**Affiliations:** Department of Chemistry, Portland State University, Portland, Oregon, United States of America; Hong Kong Polytechnic University, HONG KONG

## Abstract

E-cigarette devices are wide ranging, leading to significant differences in levels of toxic carbonyls in their respective aerosols. Power can be a useful method in predicting relative toxin concentrations within the same device, but does not correlate well to inter-device levels. Herein, we have developed a simple mathematical model utilizing parameters of an e-cigarette’s coil and wick in order to predict relative levels of e-liquid solvent degradation. Model 1, which is coil length/(wick surface area*wraps), performed in the moderate-to-substantial range as a predictive tool (R^2^ = 0.69). Twelve devices, spanning a range of coil and wick styles, were analyzed. Model 1 was evaluated against twelve alternative models and displayed the best predictability. Relationships that included power settings displayed weak predictability, validating that power levels cannot be reliably compared between devices due to differing wicking and coil components and heat transfer efficiencies.

## Introduction

The ongoing development and popularity of electronic cigarettes have challenged scientists and regulators. Issues faced by researchers include the rapidly evolving devices and e-liquid formulations, as well as a lack of standardized testing methods. These factors have exacerbated the significant inter-laboratory variability in reported e-cigarette aerosol toxin levels [1–3]. For example, Beauval et al compared carbonyl emissions from twenty different e-cigarette studies and found reported ranges from 2–342,220 ng/puff for formaldehyde and 0.3–135,468 ng/puff for acetaldehyde [4]. Factors such as puff volume [4], e-liquid consumed [5] and power output [6] are known to correlate to toxin levels intra-device; however, methods for understanding inter-device levels are still needed. For instance, ten times lower toxin levels were reported in the aerosols produced by an e-cigarette operated at 50 W compared to a different e-cigarette operated at 10 W [7].

**Fig 1:**
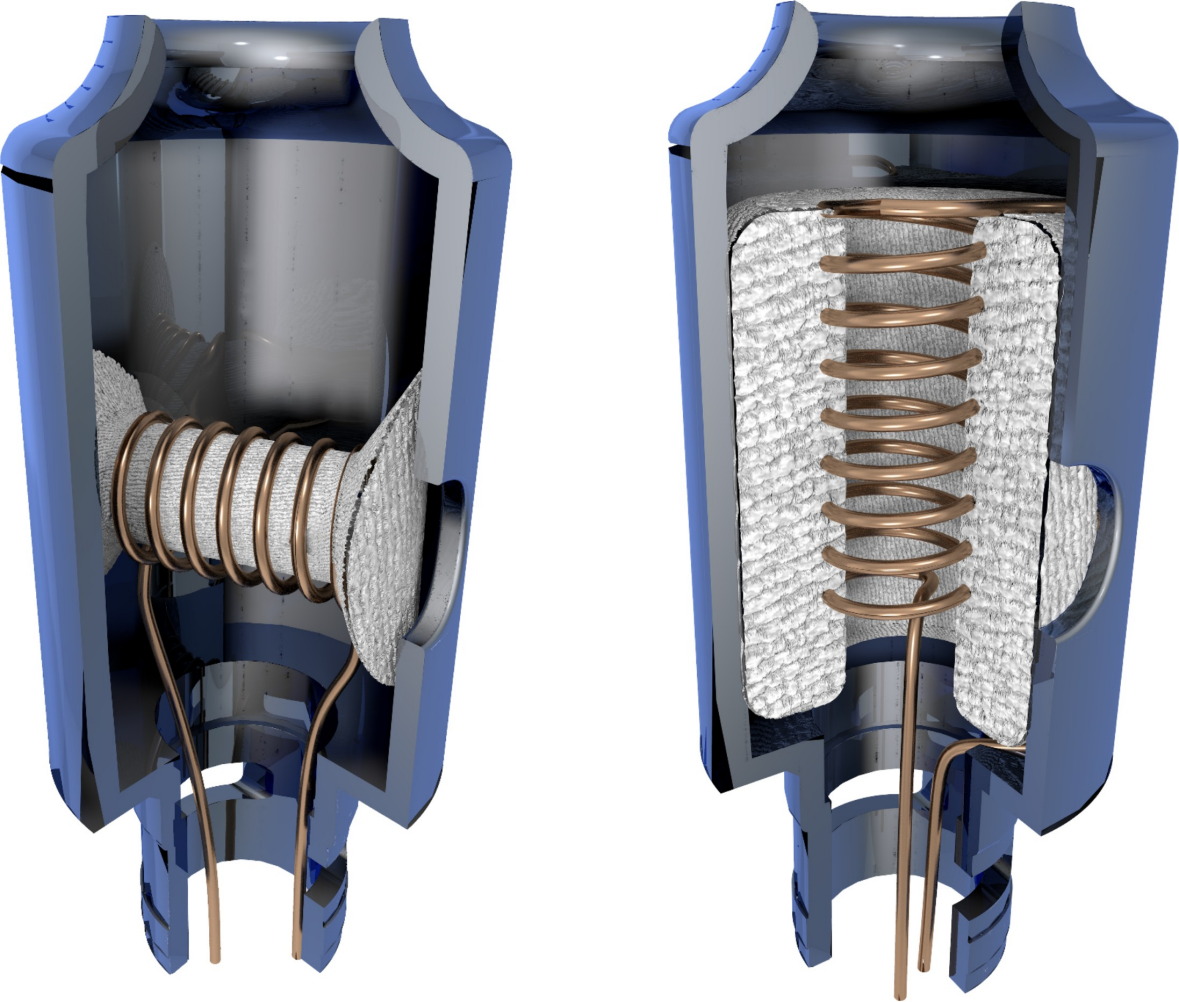
**Depiction of horizontal (left) and vertical (right) coil orientations.** The vertical coil has a surrounding wick.

E-cigarette products are wide-ranging, affording device-specific levels of toxic emissions [8–10]. For example, Saliba et al. reported that catalysis of the degradation of e-liquid solvents can occur in a manner dependent on the type of coil material, resulting in enhanced toxin levels [11]. Other specific coil properties are still under investigation [12–14]. One study showed that total carbonyl yields are proportional to device power (Watts) divided by coil surface area [15]. According to the researchers, additional study is needed beyond the two tank models examined to date. In a related investigation, an inverse relationship was identified between coil volume and nicotine aerosol yield [16]. Coil volume was defined as the cylinder formed by the wick surrounded by the coil; which, by definition, limits utility to only horizontal coils (Fig 1).

**Fig 2:**
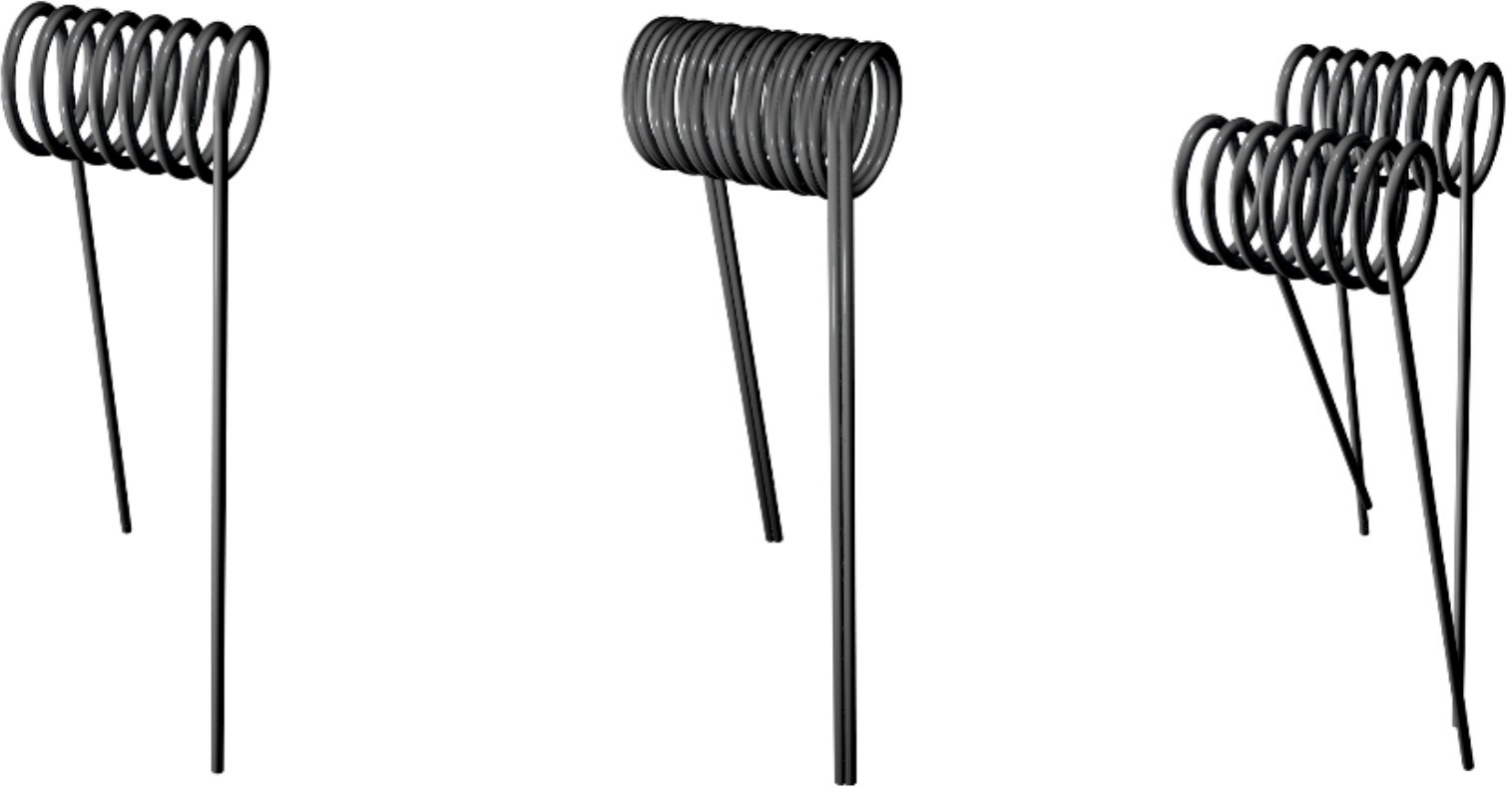
**Illustration of single (left), parallel (middle) and dual coils (right).** The parallel and dual coil contains two identical wires; however, the dual coils are wrapped individually with respective wicks. Each style can be oriented vertically or horizontally.

E-cigarette atomizers contain a heating coil and a porous wick with various materials, designs and styles. The traditional coil contains a helical wire made of Kanthal, nichrome or stainless steel that is paired with a wick made of cotton or silica. The coil and wick can be oriented vertically or horizontally (Fig 1). The styles of coil include single, parallel and dual (Fig 2).

Propylene glycol and glycerol (PG/GL) degrade through excess heat; therefore, an e-cigarette coil that has a consistent supply of e-liquid and uniform heating will produce less toxins. It has been shown, for instance, that there is an inverse relationship between the efficiency of e-liquid consumption and toxic carbonyl aerosol levels [17]. According to Wang et al., the production of carbonyls can be substantially increased as the temperature of the e-liquid (i.e., PG, GL, or mixture) increases. Formaldehyde and acetaldehyde formation from PG and GL started at 215°C and increased exponentially as temperature of the liquid increased. At a temperature of 318°C, formaldehyde, acetaldehyde, acetone and acrolein were generated [18]. In the current study, we expand on our previous report on e-cigarette wicking properties [7], wherein we had shown that the stability of the wick temperature during aerosolization served as a predictor of the degree of PG/GL degradation.

Herein, we focus on coil design and wicking in modulating toxin production. A new mathematical model for predicting relative toxin levels based on reasonably simple coil and wick measurements is derived. It can be used as a means for regulators and manufacturers to predict relative emissions and health risks associated with specific e-cigarette design features.

## Methods

### Electronic cigarette devices

Twelve unique coil atomizers (EC1-12) were used for aerosolization (S1 Table). Each device was powered by a SMOK® Alien 220W variable voltage/variable wattage/temperature control (VV/VW/TC) battery. Due to the wide range of resistances it would not be recommended to test each atomizer at the same power output; therefore, each atomizer was tested at the highest wattage of the manufacturer specified range. The higher power output was chosen to better insure collected carbonyl levels were above the LOQ for all devices.

#### E-liquid preparation and avoidance of dry coils and burnt e-liquid

Each device was filled with e-liquid to the highest level according to manufacturers’ recommendation. A mixture of PG/GL (50% by volume, v/v) was prepared in house from ACS-grade PG and GL and used for all sample testing. E-liquid consumed was calculated by weighing the e-cigarette tank before and after each session.

New coils were used for each session. Before sample collection, each coil was primed with five “warm-up” puffs starting at a lower wattage and increasing evenly until the target wattage was reached by the fifth puff. Alternatively, JUUL e-cigarettes do not afford control of power output; therefor, each coil was primed with 5 puffs at full charge. If the e-liquid or atomizer exhibited an unusual smell after the session, indicating burnt e-liquid, the sample was discarded. If the e-cigarette did not produce visible aerosol, indicating improper e-liquid supply to the coil, the sample was also discarded.

#### JUUL pod e-liquid replacement

JUUL e-cigarettes are commercially available with replacement cartridges pre-filled with e-liquid containing PG, GL, nicotine, flavorants and other additives. In order to minimize variables which could affect our analysis, the cartridges needed to be emptied and filled with the prepared 1:1 PG/GL e-liquid. Unused JUUL pods were emptied of e-liquid, washed by sonication with water followed by sonication with methanol. All components were allowed to dry at minimum 24 hours, then refilled with PG/GL and reassembled.

### Puffing regime

A CH Technologies single cigarette smoking machine (SCSM-STEP) was used. The smoking machine was set to the CORESTA program with a square shape puff profile, a 3 s puff period, 30 s puff interval and a 55 mL puff volume. This program was used for all samples collected. Each e-cigarette was tested at minimum in triplicate.

### DNPH solution

DNPH solution was prepared in accordance with the CORESTA standardized method from DNPH stock solutions. DNPH was purified via recrystallization [19]. Approximately 3 g DNPH hydrate was weighed and added to 62.0 mL EtOH and warmed with magnetic stirring agitation. 80.0 mL EtOAc was added slowly with heat and stirring until all of the DNPH was dissolved. The warm solution was vacuum filtered and transferred to an Erlenmeyer flask and cooled overnight. The DNPH crystals were isolated using vacuum filtration. The crystals were placed in a desiccator to protect from moisture. Recrystallized DNPH (0.805 g) was added to 175 mL acetonitrile (MeCN) and 75 mL of H_2_O containing 3.5 mL phosphoric acid (85%). Fresh 250 mL batches of DNPH stock solution were prepared weekly and stored in amber flasks at room temperature.

### Aerosol collection for HPLC analysis

The aerosol produced was passed through an impinger containing 20.00 mL DNPH solution. 40 puffs were collected. After each session, the DNPH solution was collected into an amber vial and analyzed within 4 hours.

### Analysis by HPLC-UV

DNPH samples were analyzed and quantified using a Waters® 1525 Binary HPLC Pump and a Waters 2996 Photodiode Array Detector. Analysis conditions: two SUPELCOSIL C-18 (25 cm × 4.6 mm, 5 μm particle size) columns connected in series inside a column heater at 40°C. The mobile phase comprised of MeCN/H_2_O with a gradient system as follows: 0 min. 60/40; 7 min. 60/40; 25 min. 100/0, at a combined flow rate of 1 mL/min, with 360 nm detection wavelength. The sample injection volume was 20 μL.

### Rebuildable coil

A Zeus dual RTA by Geekvape® was used with Kanthal A-1 wire (30 gauge) and Wick N’ Vape cotton bacon (v2). A dual coil build compromised of two wires of 98.04 ± 0.05 mm length wrapped 8 times independently. Two cotton wicks weighing 24.82 ± 5.09 mg each were fit through the dual coils, one wick per coil. A new coil was built for each run, and ten puffs were performed and discarded at the target power output of 65W before the collection of aerosol to be analyzed by HPLC-UV.

### Statistical rigor

Each e-cigarette (EC1-12) was tested at minimum in triplicate, using a new coil for each session. Post analysis a Grubbs outlier test was performed and subsequent outliers were identified and removed. These outliers could account for burnt coils, improper ohmage readings from the power battery and irregularities in the coil build. To verify model 1 is a good predictor, other coil and wick calculations were analyzed in the same fashion. Of the ten tested, model 1 performed the best when comparing the experimental values to the predicted values. A selected number (10 of 13) of alternative models are included in the Supporting Information.

## Results and discussion

### Developing a general mathematical model

Wicking controls the supply of e-liquid to the coil and enables heat transfer from the coil to the e-liquid. The wick’s outer surface area influences the amount of e-liquid that can be absorbed at any given point in time. A wick with a relatively large surface area will allow a larger area of e-liquid to absorb thermal energy from the heating coil, therefore reducing e-liquid temperature and typically leading to a faster cooling post latent heat gain [20].

Heat transfer is known to be a function of surface area of a metal [21]. The larger the surface area, the more heat can be applied to a system. However, within the e-cigarette atomizer, some of the coil’s thermal energy is being absorbed by the e-liquid soaked wick, while some of the thermal energy is transferred to the air passing over the coil. Therefore, the coil surface area would lead to an inaccurate estimation of thermal energy transferred to the e-liquid, whereas coil length is a more representative indicator.

The number of times the coil is wrapped or turned defines the contact of thermal energy transfer between the coil and the wick. A higher number of coil wraps will enable more even thermal energy distribution throughout the wick [22], thereby reducing “hot spots”, lowering the temperature of the e-liquid and decreasing the degradation of e-liquid components. It is important to note within parallel style coils there is not space between each wire which increases the amount of thermal energy being applied at each contact point of the wick, potentially increasing the temperature of the area. Additionally, in horizontal style coils, wherein the coils are wrapped around the wick material, an increase in turns while maintaining coil length will lead to a decrease in the wick outer surface area, thereby altering wicking efficiency. In order to account for the variable effects of wick surface area (SA), coil wraps and coil length on wicking efficiency, we developed a model (1) to predict relative toxin emission levels in varying e-cigarette brands and devices.

$$aerosol\;carbonyl\;toxin\;levels\propto \frac{coil\;length\;(mm)}{wick\;surface\;area\;({mm}^{2})\times (n)wraps}$$ (1)

            *(n) wraps = the number of coil turns*

### Using model 1 to analyze relative levels of carbonyls produced by nine different e-cigarettes

**Fig 3:**
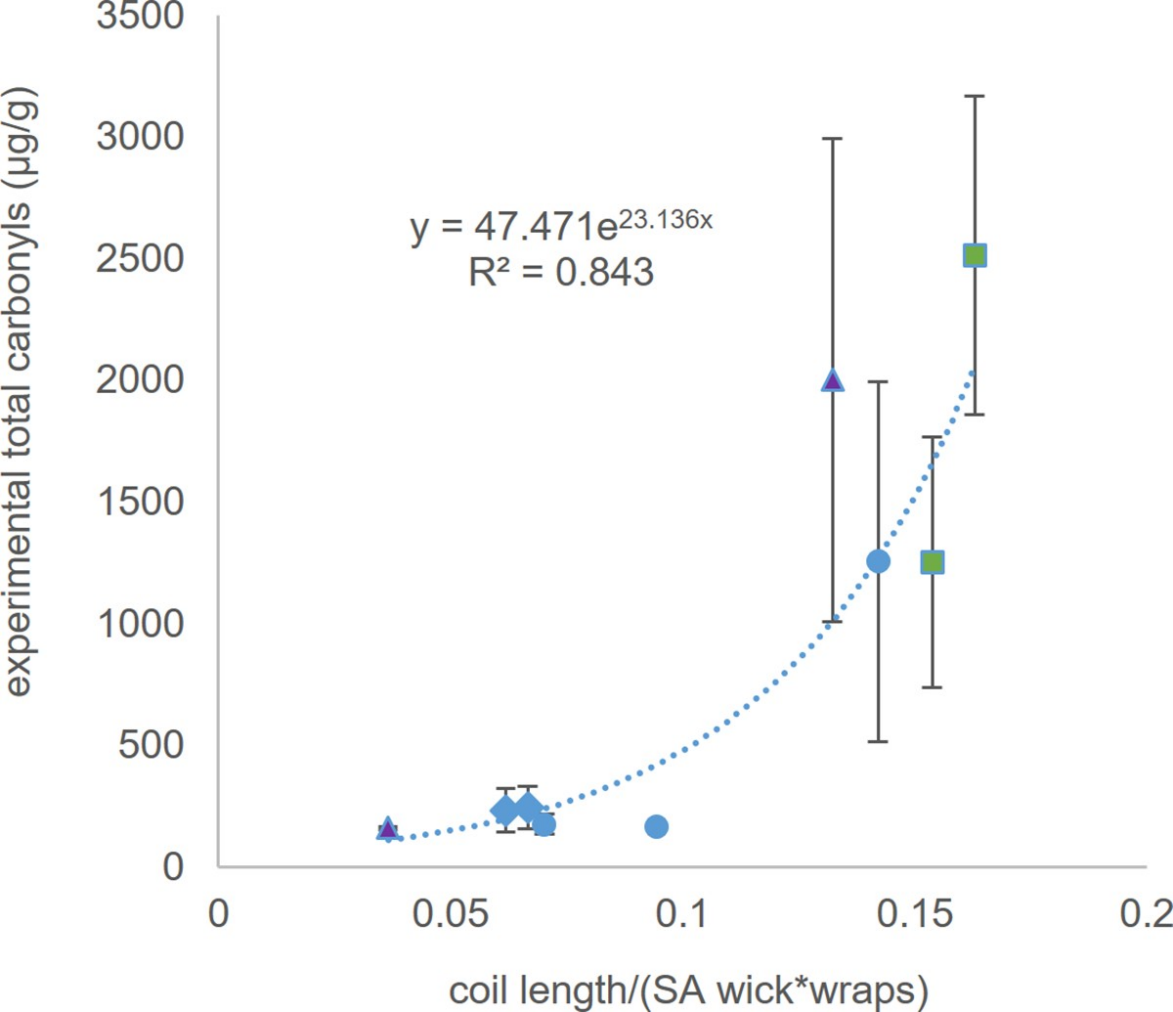
Total carbonyl emissions per e-liquid consumed as a function of coil length per the product of outer surface area of wick and coil wraps (1). Dashed line represents best fit regression (R^2^ = 0.843). Each carbonyl was analyzed by HPLC (see methods section). Error bars represent one standard error. The larger error bars associated with the e-cigarettes that produce high levels of toxins are expected based on previous literature [4,25]. Higher concentrations of carbonyls suggest inefficient wicking that produce a higher degree of error exacerbated by intra-device inconsistencies. EC1-9 are various brands of e-cigarettes with differing wicks, coils and coil styles (see Supporting Information). Blue circle symbols represent devices with single coil style, green square symbols represent devices with parallel coil style, and purple triangle symbols represent devices with dual coil style.

To initially test model 1, nine different devices (EC 1–9), covering all common orientations and styles (Figs 1 and 2 and S1 Table) were used to aerosolize 1:1 PG/GL (% v/v) in their respective commercial e-cigarettes without modification. The model 1 parameters were plotted along with the measured concentrations of six target carbonyls produced via e-liquid solvent degradation: formaldehyde, acetaldehyde, acetone, propanal, butyraldehyde and benzaldehyde. Their levels were monitored using the standard EPA method with an impinger of 2,4-dinitrophenylhydrazine (DNPH) and HPLC-UV [23]. An exponential regression analysis was obtained between model 1 and the experimental carbonyl concentration levels measured (Fig 3). The data was analyzed as an exponential relationship due to the exponential behavior for enthalpy of vaporization, as is consistent with the Clausius-Clapeyron equation [24].

Interestingly, Fig 3 shows two clusters of e-cigarettes, class 1 with lower toxin levels and smaller error bars and class 2 exhibiting higher toxin levels and larger error bars. This data reflects the fact that devices that show more e-liquid degradation to toxic aldehydes have less efficient and relatively unreliable and inconsistent wicking properties. The model is very effective at delineating between these two classes of e-cigarettes. Importantly, each classification is not a function of any one type of component. For example, class 1 and class 2 both contain devices with different style coils (e.g., horizontal and vertical, single and dual).

### Testing the predictive nature of model 1 using twelve different e-cigarettes

**Fig 4:**
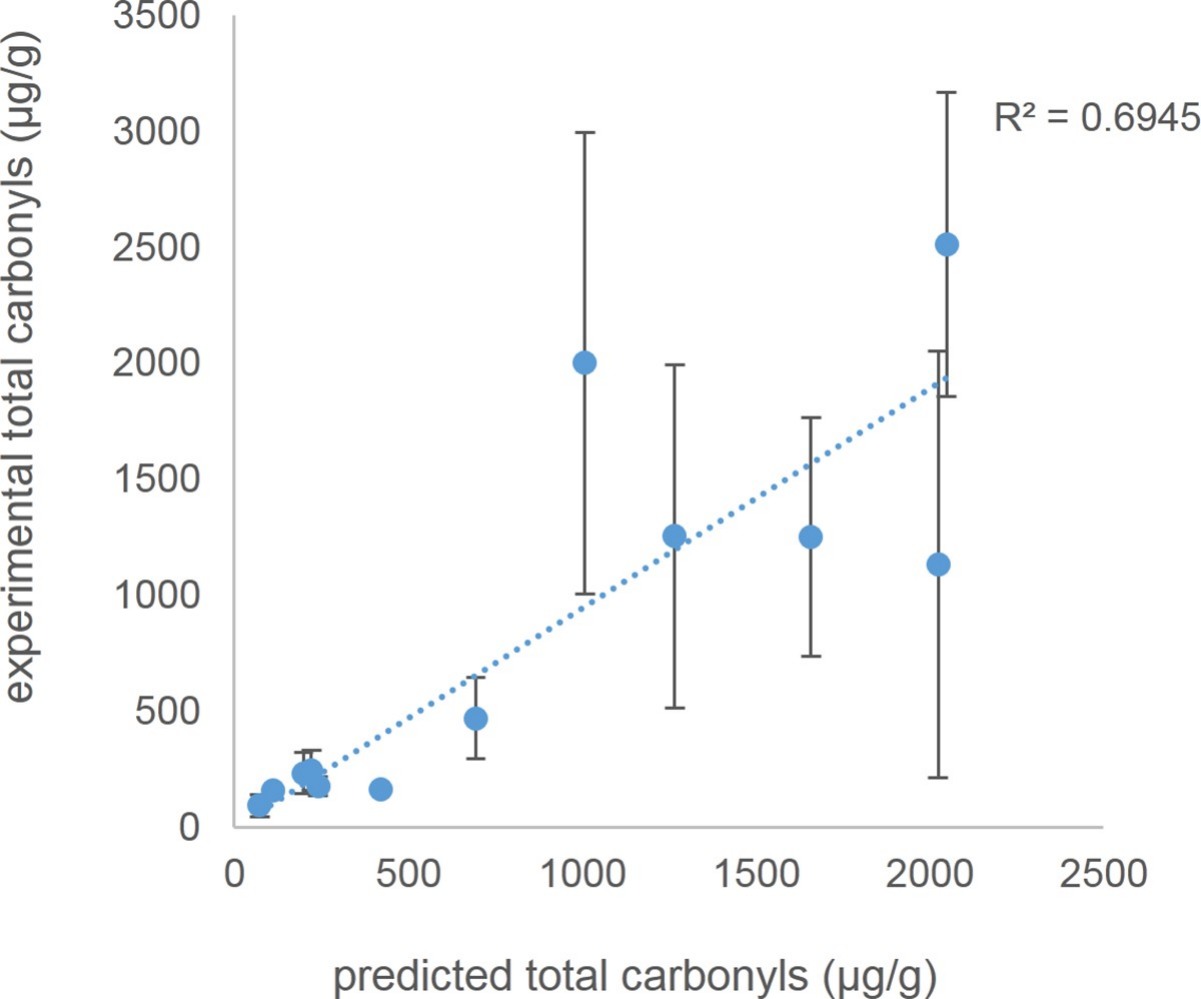
Experimental versus exponential fitted values of the regression analysis of EC1-9 with the experimental values of EC1-12. This demonstrates model 1 performs in the moderate-to-substantial range as a predictive tool (R^2^ = 0.6945) [26]. Dashed line represents best fit regression. Error bars represent one standard error. EC1-12 are various brands of e-cigarettes with differing wick, coil material and coil style (see Supporting Information).

To support the hypothesis that model 1 can be used to potentially predict relative toxin levels of e-cigarette aerosols three additional e-cigarettes (EC10-12) were used to aerosolize 1:1 PG/GL. The predicted carbonyl concentration levels of all twelve e-cigarettes were calculated based on the equation of the exponential regression from the initial nine e-cigarettes (Fig 3). The comparison of the experimental and predicted carbonyl concentration levels gave a moderate-to-substantial predictive accuracy [26] (R^2^ = 0.6945) (Fig 4); the classification of substantial, moderate and weak correlations based on R^2^ thresholds is based on Hair et al. [26].

### Testing alternative models’ predictability

We also evaluated alternative models, such as those including power levels, to compare their predictive measures of aerosol components [15,16]. It is well-known that an increase in power levels will increase aerosol toxin levels within the same device [27]. However, the same power levels cannot be reliably compared between devices due to differing wicking and coil components and heat transfer efficiencies. In order to address this issue to choose a power level that would afford inter-device consistency, we used the high value of the manufacturers’ recommended range of power settings for each device studied in this work. Alternately, the method of analyzing total carbonyl levels normalized by mass e-liquid consumed could inherently account for power [28,29].

**Fig 5:**
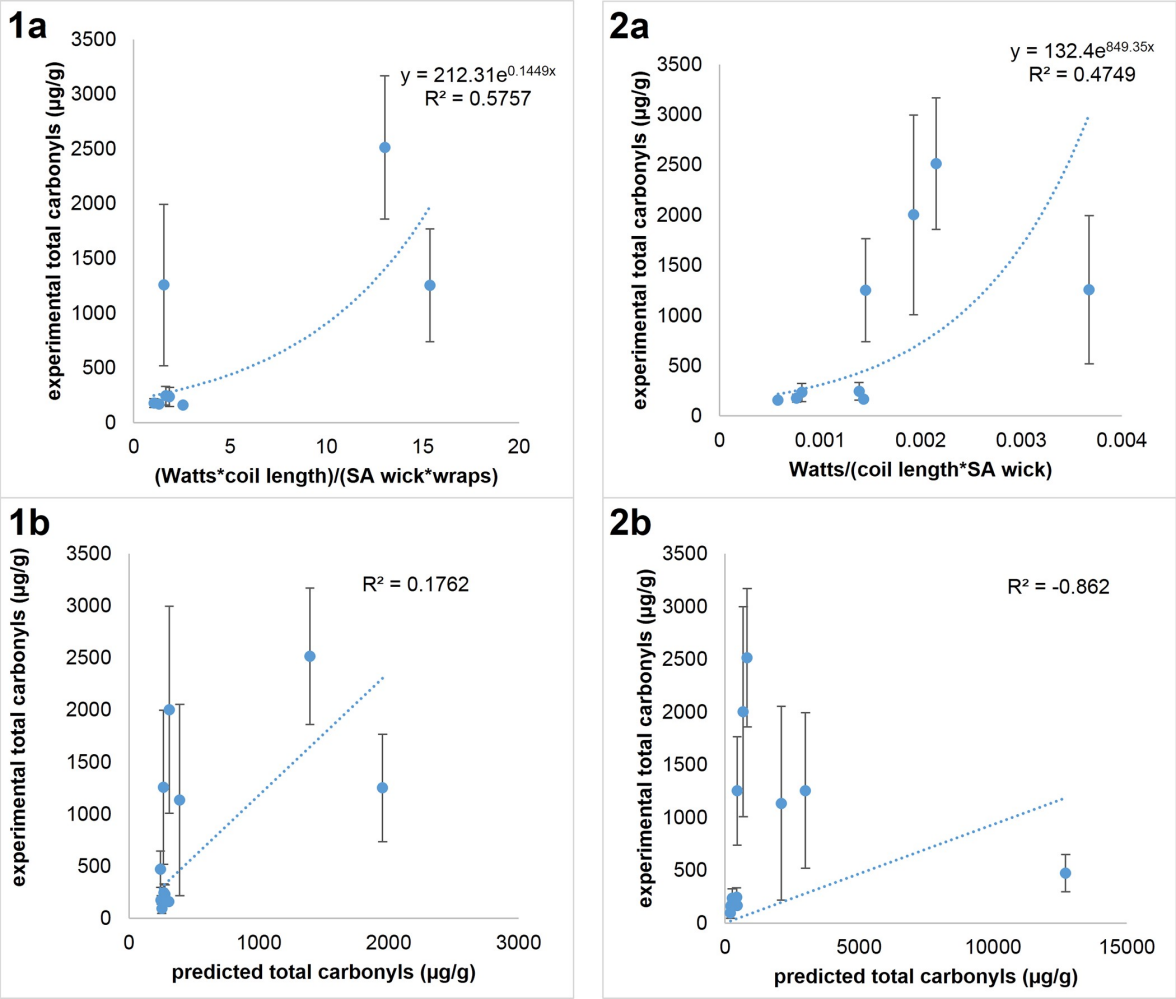
Analyzing alternative models as predictors of measured toxins. Graph **1a** shows the regression analysis of EC1-9 using (Watts*coil length)/(SA wick*wraps). Graph **1b** displays a weak predictability (R^2^ = 0.1762) of EC1-12 using the equation of the regression analysis from **1a**. Graph **2a** shows the regression analysis of EC1-9 using Watts/(coil length*SA wick). Graph **2b** displays a weak predictability (R^2^ = -0.862) of EC1-12 using the equation of the regression analysis from **2a**.

The results of alternative models indicate that those that incorporate power settings as variables display weak predictability [26] (Fig 5) when comparing multiple brands and devices. Twelve alternative models were investigated, as shown in Fig 5 as well as in the Supporting Information.

## Conclusion

A straightforward model based on e-cigarette coil and wick measurements is described to enable the efficient prediction of the relative degree of e-cigarette solvent degradation between varying brands. Testing twelve different e-cigarettes, encompassing a variety of coil and cotton styles, atomizer measurements (model 1) correlated well with experimental concentrations (R^2^ = 0.843) of six target carbonyls. Comparison of the predicted values of the regression analysis of EC1-9 with the experimental values of EC1-12 demonstrates that model 1 performs in the moderate-to-substantial range as a predictive tool (R^2^ = 0.6945). It clearly enabled the separation of devices into those generating lower toxin levels with greater reproducibility versus those producing greater aerosol toxin levels with greater variability. Model 1 exhibited the best relationship out of the 13 models tested. Interestingly, models that contained power variables produced a weak predictability of relative total carbonyl concentrations.

One limitation of this study is the testing of only six gas-phase carbonyls. There may be significant quantities of other degradants present in gas phase as well as particle phase [5], however, it is unlikely to change the basic observations of this study. There are also other parameters that can influence e-liquid degradation such as vaping topography, catalysis and specific additives [30]. However, model 1’s simplicity renders it potentially useful in predicting relative levels of carbonyl toxins across widely varying device generations and styles. A second limitation is only twelve e-cigarette devices were tested. Each device design varies, and there may be new designs commercially available that challenge model 1’s effectiveness. The development of model 1 was established by analyzing the relationships of seven different variables. The preceding explanation of coil length, wick surface area and number of turns influence on toxin production may not be irrefutable; however, this relationship displayed the best predictability of relative aerosol carbonyl levels according to experimental analysis.

## Supporting information

S1 Table E-cigarette identification and coil style of twelve different coils. (PDF)

S2 Table Coil and wick measurements of twelve different e-cigarettes, each measured at minimum in triplicate. (PDF)

S3 Table Average concentrations of 6 target carbonyls generated from twelve e-cigarettes tested. (PDF)

S4 Table Average mass e-liquid consumed during each independent collection from twelve coils tested. Values represented as grams e-liquid per single puff.
(PDF)

S1 Appendix Calibration curves for HPLC analysis. (PDF)

S2 Appendix Alternative models were tested and compared to model 1. (PDF)

S3 Appendix Additional statistical analyses. (PDF)
