# Supplementary material for: A simple predictive model for estimating relative e-cigarette toxic carbonyl levels
Source: PLoS One. 2020 Aug 26;15(8):e0238172. doi: 10.1371/journal.pone.0238172 (PMC7449472; doi:10.1371/journal.pone.0238172)
Supplement: S1 Table — (PDF) [file pone.0238172.s001.pdf]

Table S1. E-cigarette identification and coil style of twelve different coils

E-cigarette brand name	Reference number	Coil orientation	Coil style	Resistance (Ω)	Manufacturer recommended setting (W)	Power level tested (W)	Coil wire type
SMOK Baby Q2	EC1	vertical	parallel	0.4	40-80	80	Kanthal
SMOK Baby X4	EC2	vertical	dual	0.15	30-70	70	Kanthal
Eleaf iJust 2 Mini	EC3	vertical	parallel	0.5	30-100	100	Kanthal
Joyetech Cubis	EC4	vertical	single	0.5	15-30	30	stainless steel
Aspire Nautilus Mini	EC5	vertical	single	1.8	10-14	14	Kanthal
Kanger Protank 2	EC6	horizontal	single	2.2	N/R	11	nichrome
Kanger Subtank Mini	EC7	vertical	single	1.2	7-15	15	nichrome
Halo Triton 2 (0.75 Ω)	EC8	vertical	single	0.75	8-25	25	Kanthal
Halo Triton 2 (1.5 Ω)	EC9	horizontal	dual	1.5	8-20	20	Kanthal
Geekvape Zues Dual RTA	EC10	horizontal	dual	1.38	N/A	65	Kanthal
JUUL	EC11	horizontal	single	2.0	N/A	8	nichrome
Kanger Subtank Mini (26W)	EC12	vertical	single	1.2	10-26	26	nichrome

N/R: not reported. For e-cigarettes whose power rangers were not reported by the manufacturer, user's self-reported ranges from online sources were used.
N/A: not applicable.
