# Supplementary material for: A simple predictive model for estimating relative e-cigarette toxic carbonyl levels
Source: PLoS One. 2020 Aug 26;15(8):e0238172. doi: 10.1371/journal.pone.0238172 (PMC7449472; doi:10.1371/journal.pone.0238172)
Supplement: S2 Table — (PDF) [file pone.0238172.s002.pdf]

Table S2. Coil and wick measurements of twelve different e-cigarettes, each measured at minimum in triplicate. All measurements were taken with a high precision caliper post sample collection. Not all measurements were used in the discussion of this study.

E-cigarette	Reference number	Coil length (mm)	Coil diameter (mm)	Cotton length (mm)	Cotton diameter (mm)	Coil length (coiled, mm)	Coil diameter (coiled, mm)	Inner coil diameter (mm)	Number of wraps (n)	Surface area of cotton (mm2)
SMOK Baby Q2	EC1	154.04	0.46	7.44	9.8	8.14	6.95	6.03	4	229.06
		155.19	0.47	8.27	9.26	6.9	6.94	6	4	240.58
		157.71	0.45	7.91	9.96	7.29	6.93	6.03	4	247.51
SMOK Baby X4	EC2	223.94	0.36	15.52	5.46	5.89	4.14	3.42	12	532.4
		231.28	0.37	15	5.41	6.26	3.81	3.07	12	509.9
		230.82	0.37	14.54	5.66	6.39	3.84	3.1	12	517.1
		232.40	0.36	14.52	5.89	6.4	3.66	2.94	12	537.4
Eleaf iJust 2 Mini	EC3	294.16	0.41	9.44	8.1	9.55	5.18	4.36	8	240.2
		289.38	0.30	9.59	7.74	9.84	5.17	4.57	8	233.2
		294.34	0.37	9.57	7.04	9.62	5.12	4.38	8	211.7
		284.34	0.34	9.53	8.93	9.97	5.05	4.37	8	267.4
Joyetech Cubis	EC4	153.14	0.30	10.43	7.38	9.16	2.69	2.09	10	241.8
		148.00	0.30	10.28	7.13	9.02	2.85	2.25	10	230.3
		151.37	0.30	10.78	7.7	9.2	2.63	2.03	10	260.8
Aspire Nautilus Mini	EC5	93.47	0.22	6.54	5.68	5.23	2.52	2.08	9	116.7
		92.24	0.12	6.27	5.1	6.09	2.5	2.26	9	100.5
		92.33	0.18	6.01	5.33	5.95	2.47	2.11	10	100.6
Kanger Protank 2	EC6	47.11	0.13	9.89	1.56	3.4	2.01	1.75	5	66.2
		46.61	0.14	10.34	1.54	3.42	1.9	1.62	5	64.6
		44.66	0.14	10.24	1.54	3.01	1.98	1.7	5	64.0
Kanger Subtank Mini (15W)	EC7	113.76	0.22	6.95	7.85	5.47	3.2	2.76	9	171.4
		107.33	0.20	8.11	7.87	6.94	3.13	2.73	9	200.5
		111.37	0.20	7.2	7.28	7.37	3.15	2.75	9	164.7
		111.48	0.21	7.38	7.39	6.57	3.17	2.75	9	171.3
Halo Triton 2 (0.75 Ω)	EC8	97.75	0.29	7.75	7.25	7.18	3.38	2.8	8	176.5
		97.91	0.30	8.04	7.65	7.54	3.43	2.83	8	193.2
		98.32	0.29	7.76	7.35	6.25	3.4	2.82	8	179.2
		97.88	0.28	7.7	7.76	7.34	3.41	2.85	8	187.7
Halo Triton 2 (1.5 Ω)	EC9	152.22	0.14	6.14	1.49	3.76	2.36	2.08	16	65.39
		141.64	0.13	6.54	1.69	3.69	2.29	2.03	16	77.05
		149.68	0.15	6.43	1.54	3.24	2.38	2.08	16	68.68
Geekvape Zeus RTA dual	EC10	196.00	0.25	29.1	4.01	8.15	3.32	2.62	16	733.2
		196.00	0.25	29.93	3.43	8.46	3.16	2.6	16	645.0
		196.14	0.25	27.86	3.44	7.76	3.25	2.6	16	602.17
		196.20	0.25	31.38	3.52	6.58	3.15	2.61	16	694.0
JUUL	EC11	29.28	0.13	11.86	1.13	3.69	1.57	1.31	5	42.10
		27.39	0.13	11.78	1.6	3.15	1.6	1.34	5	59.21
		30.88	0.12	11.11	1.3	N/A	1.67	1.43	5	45.37
		29.71	0.13	11.27	1.74	3.25	1.59	1.33	5	61.61
Kanger Subtank Mini (26W)	EC12	109.97	0.18	4.92	4.66	N/A	3.01	2.65	10	67.99
		114.02	0.20	5.37	4.59	6.91	3.2	2.8	9	77.43
		109.30	0.18	5.17	4.2	5.22	3.24	2.88	10	68.22
		110.98	0.17	5.16	4.64	6.56	3.16	2.82	9	75.21
