# Supplementary material for: A simple predictive model for estimating relative e-cigarette toxic carbonyl levels
Source: PLoS One. 2020 Aug 26;15(8):e0238172. doi: 10.1371/journal.pone.0238172 (PMC7449472; doi:10.1371/journal.pone.0238172)
Supplement: S3 Table — (PDF) [file pone.0238172.s003.pdf]

Table S3. Average concentrations of 6 target carbonyls generated from twelve e-cigarettes tested. The error represents one standard error of three or more independent collections.

E-cigarette	Reference number	Total carbonyls (µg/g)	Standard error
SMOK Baby Q2	EC1	2513.3	655.05
SMOK Baby X4	EC2	160.3	5.26
Eleaf iJust 2 Mini	EC3	1234.5	520.23
Joyetech Cubis	EC4	234.3	88.76
Aspire Nautilus Mini	EC5	167.2	6.20
Kanger Protank 2	EC6	1256.1	738.63
Kanger Subtank Mini (15W)	EC7	177.3	40.74
Halo Triton 2 (0.75 Ω)	EC8	245.2	86.83
Halo Triton 2 (1.5 Ω)	EC9	2002.4	993.40
Geekvape Zeus RTA dual	EC10	95.4	48.97
JUUL	EC11	472.2	175.69
Kanger Subtank Mini (26W)	EC12	1133.6	918.49

Aldehyde concentration was reported as total analytes (µg) per e-liquid consumed (g). By normalizing the analyte concentration by e-liquid consumed we eliminate the variability between individual puffs as well as individual user puffing patterns. In addition, this normalizes for power, since an increase in power output is proportional to an increase in e-liquid consumed, assuming proper wicking. This method minimizes the need for power in a predictive model, further verified by Fig S7-S16.
