# Supplementary material for: A simple predictive model for estimating relative e-cigarette toxic carbonyl levels
Source: PLoS One. 2020 Aug 26;15(8):e0238172. doi: 10.1371/journal.pone.0238172 (PMC7449472; doi:10.1371/journal.pone.0238172)
Supplement: S4 Table — Values represented as grams e-liquid per single puff. (PDF) [file pone.0238172.s004.pdf]

Table S4. Average mass e-liquid consumed during each independent collection from twelve coils tested. Values represented as grams e-liquid per single puff.

E-cigarette	Reference number	Average e-liquid consumed (g/puff)		
SMOK Baby Q2	EC1	0.03403	±	0.00204
SMOK Baby X4	EC2	0.03022	±	0.00050
Eleaf iJust 2 Mini	EC3	0.04166	±	0.00505
Joyetech Cubis	EC4	0.01658	±	0.00081
Aspire Nautilus Mini	EC5	0.01241	±	0.00047
Kanger Protank 2	EC6	0.00876	±	0.00131
Kanger Subtank Mini (15W)	EC7	0.01146	±	0.00124
Halo Triton 2 (0.75 Ω)	EC8	0.02237	±	0.00203
Halo Triton 2 (1.5 Ω)	EC9	0.01772	±	0.00376
Geekvape Zeus RTA dual	EC10	0.01078	±	0.00257
JUUL	EC11	0.00135	±	0.00152
Kanger Subtank Mini (26W)	EC12	0.02636	±	0.00273
