# Supplementary material for: A simple predictive model for estimating relative e-cigarette toxic carbonyl levels
Source: PLoS One. 2020 Aug 26;15(8):e0238172. doi: 10.1371/journal.pone.0238172 (PMC7449472; doi:10.1371/journal.pone.0238172)
Supplement: S1 Appendix — (PDF) [file pone.0238172.s005.pdf]

Appendix S1. Calibration curves for HPLC analysis.

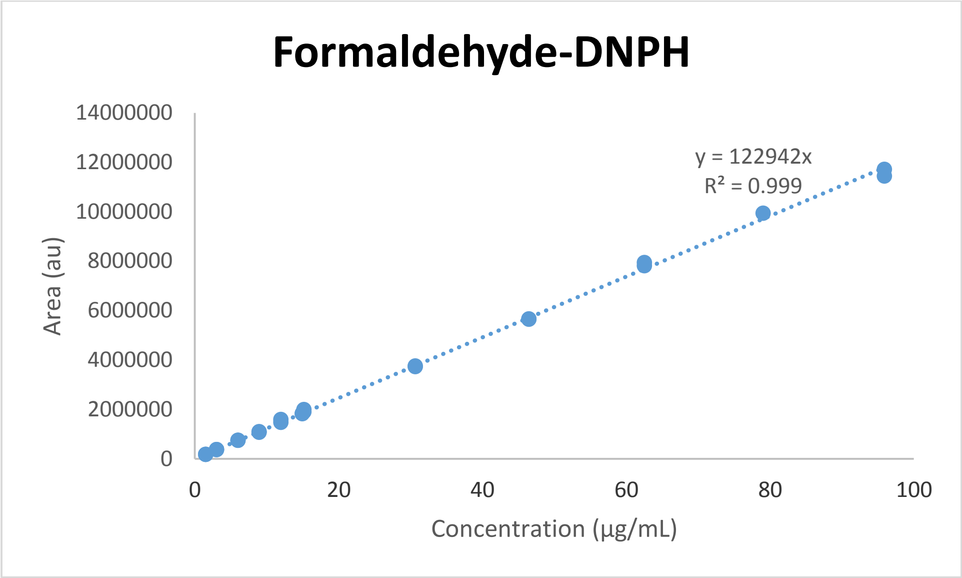

Fig S1. Formaldehyde-DNPH calibration curve for HPLC analysis. LOD was 0.105 mM.

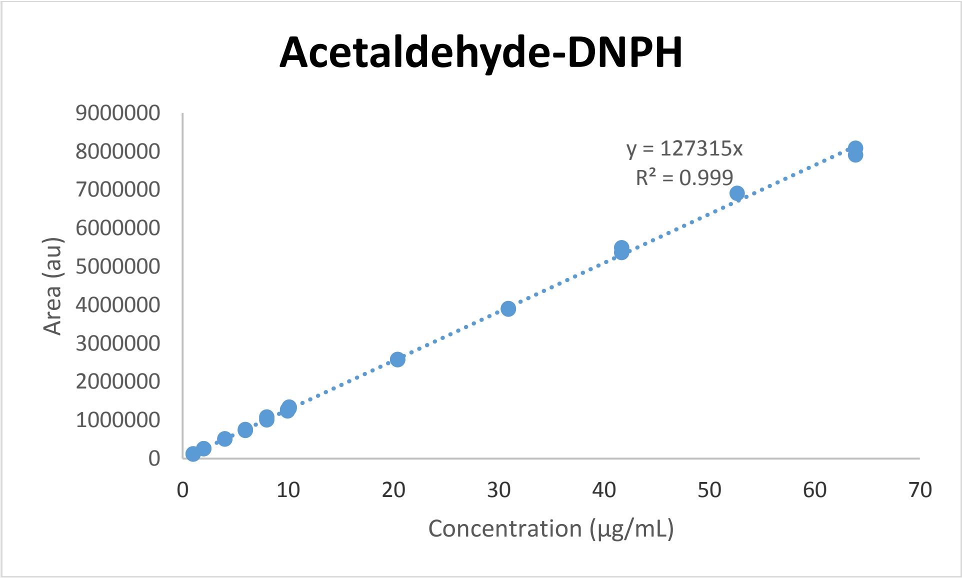

Fig S2. Acetaldehyde-DNPH calibration curve for HPLC analysis. LOD was 0.0488 mM.

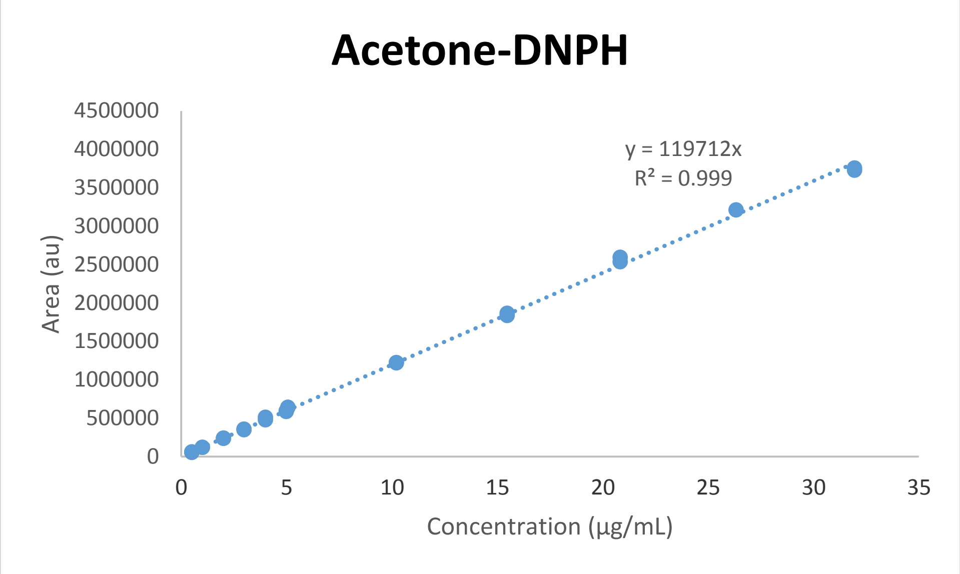

Fig S3. Acetone-DNPH calibration curve for HPLC analysis. LOD was 0.0184 mM.

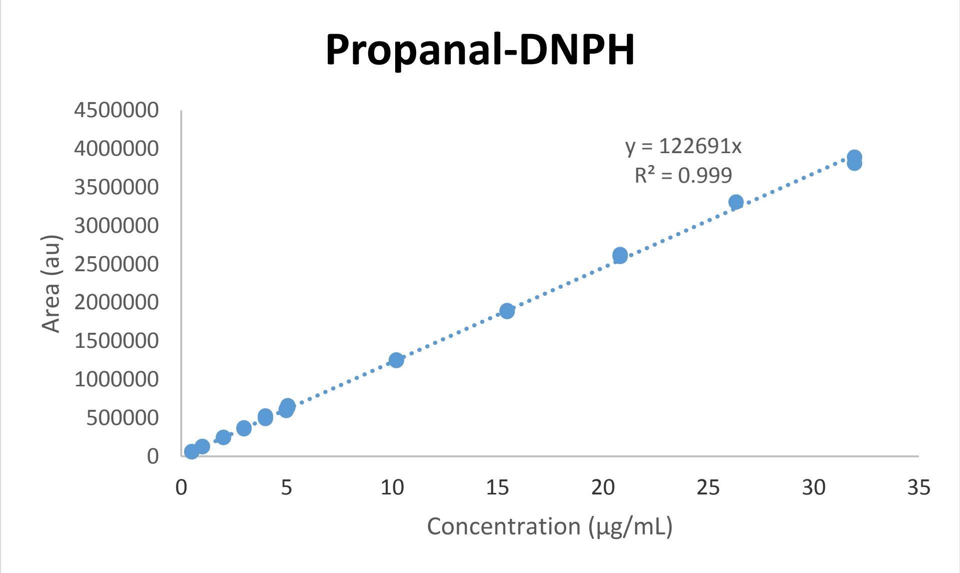

Fig S4. Propanal-DNPH calibration curve for HPLC analysis. LOD was 0.0171 mM.

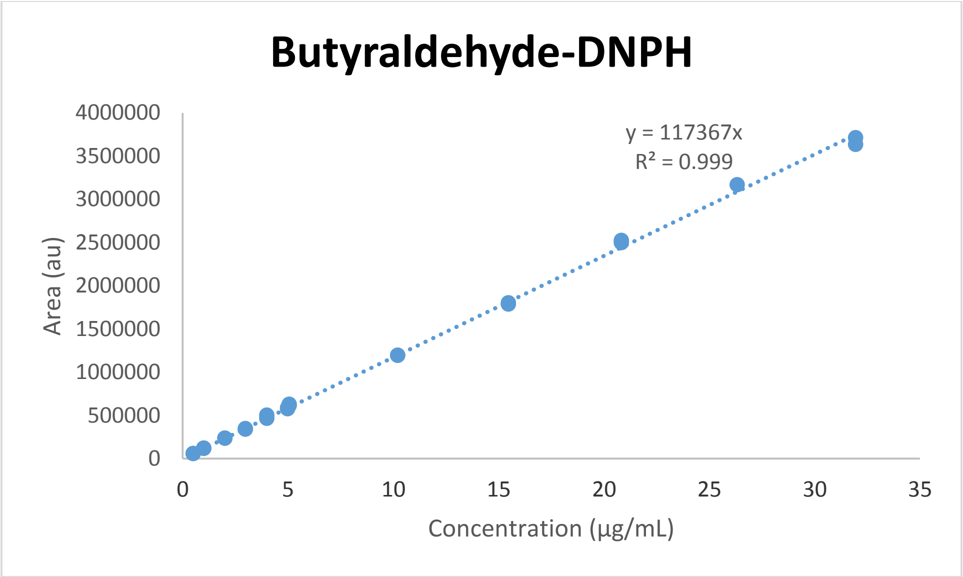

Fig S5. Butyraldehyde-DNPH calibration curve for HPLC analysis. LOD was 0.0154 mM

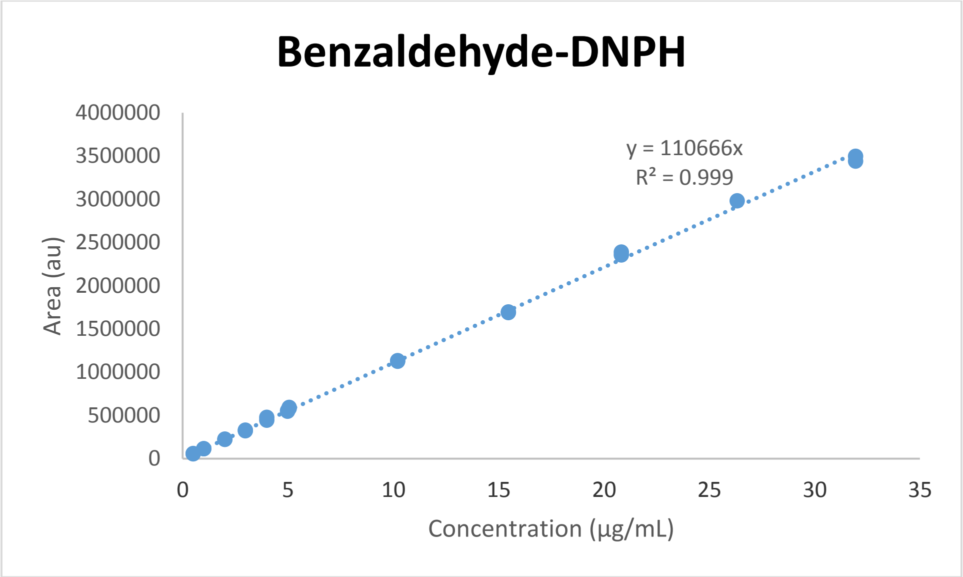

Fig S6. Benzaldehyde-DNPH calibration curve for HPLC analysis. LOD was 0.0101 mM
