# Supplementary material for: A simple predictive model for estimating relative e-cigarette toxic carbonyl levels
Source: PLoS One. 2020 Aug 26;15(8):e0238172. doi: 10.1371/journal.pone.0238172 (PMC7449472; doi:10.1371/journal.pone.0238172)
Supplement: S2 Appendix — (PDF) [file pone.0238172.s006.pdf]

Appendix S2.

Alternative models were tested and compared to model 1. Each model was plotted against experimental total carbonyl yields from EC1-9 and analyzed by exponential regression. Using the equation of the regression line predicted values of total carbonyl yields were calculated for EC1-12 and plotted against experimental yields.

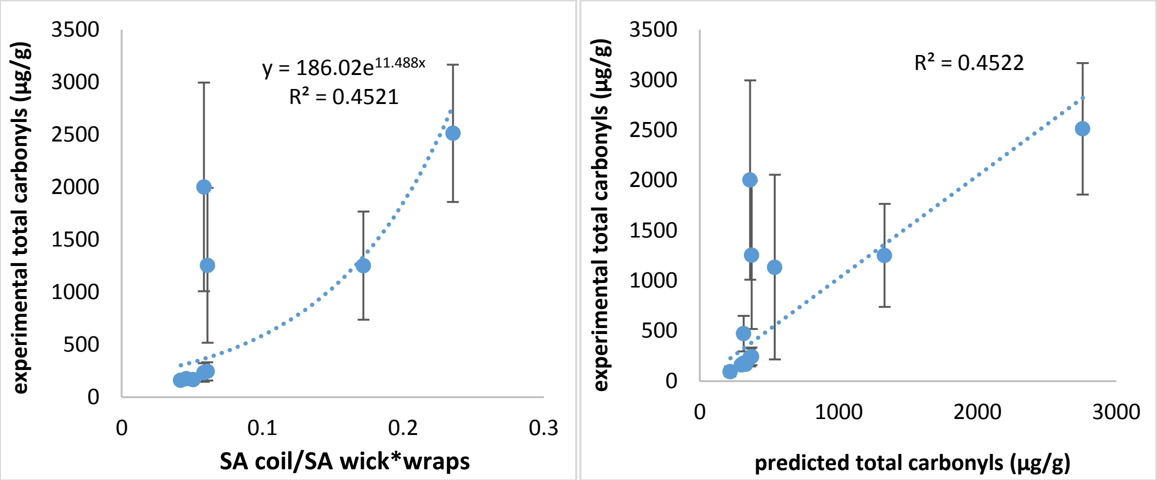

Fig S7. Regression analysis of alternative model (SA coil/SA wick*wraps) (left) and subsequent predictability (right).

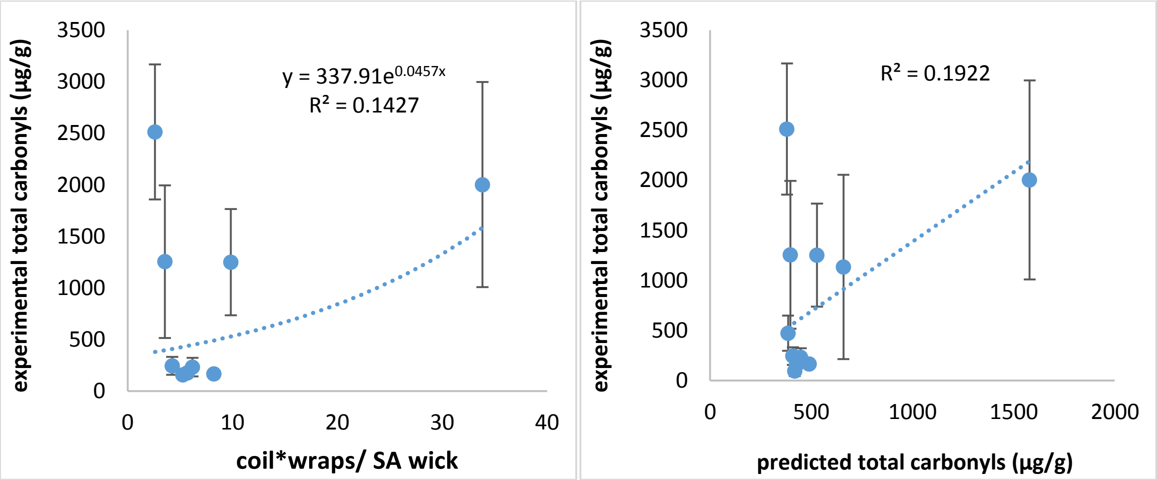

Fig S8. Regression analysis of alternative model (coil*wraps/SA wick) (left) and subsequent predictability (right).

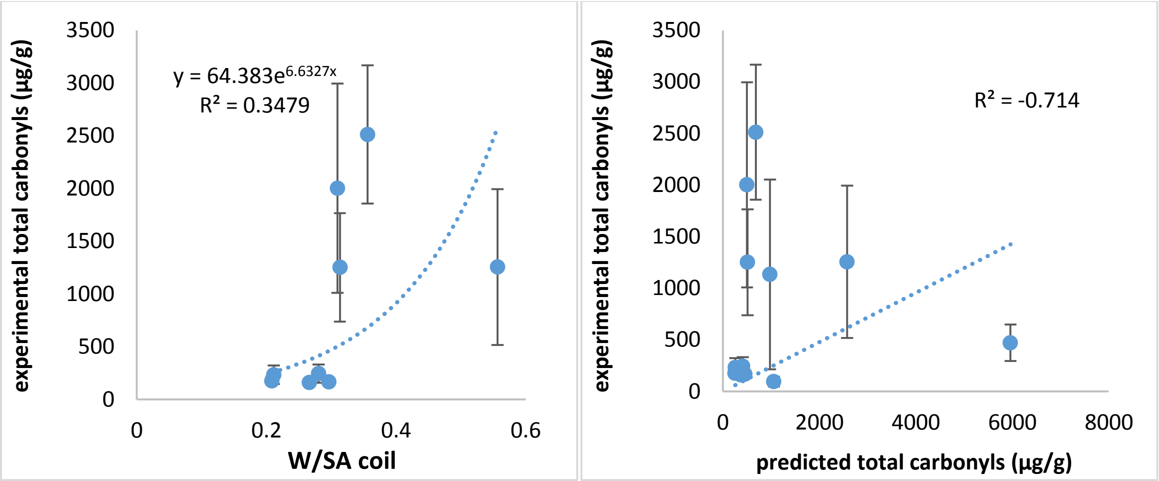

Fig S9. Regression analysis of alternative model (Watts/SA coil) (left) and subsequent predictability (right).

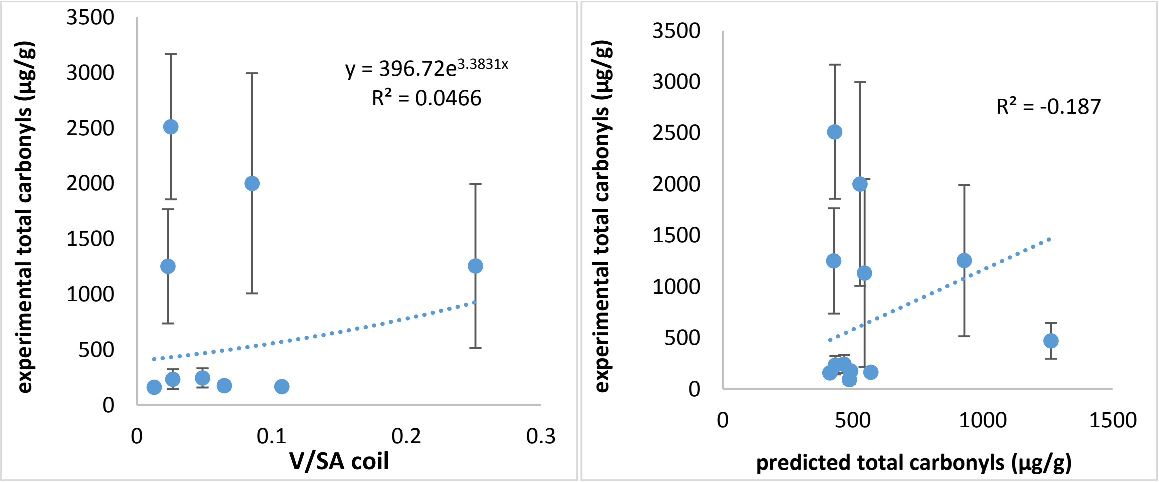

Fig S10. Regression analysis of alternative model (Volts/SA coil) (left) and subsequent predictability (right).

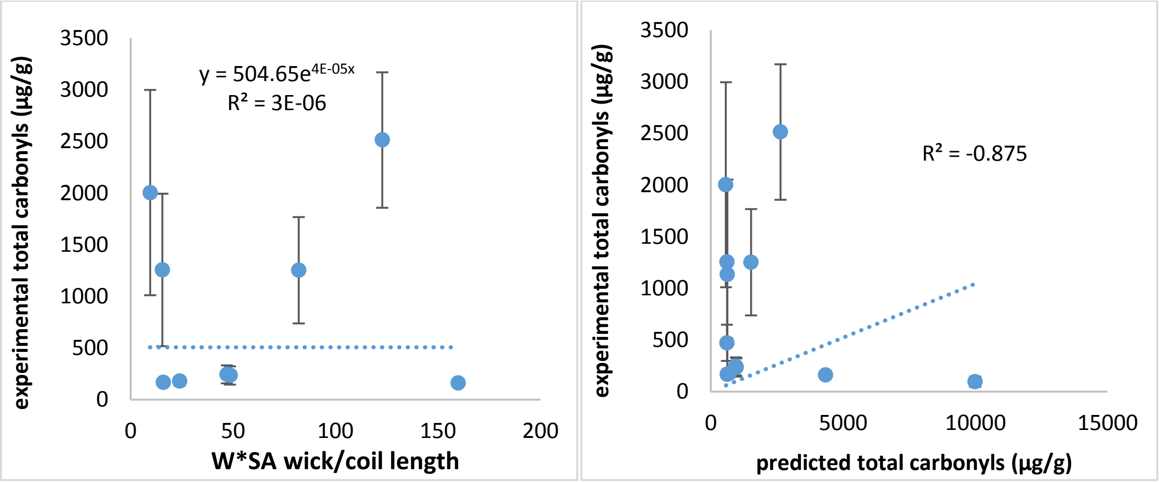

Fig S11. Regression analysis of alternative model (Watts*SA wick/coil length) (left) and subsequent predictability (right).

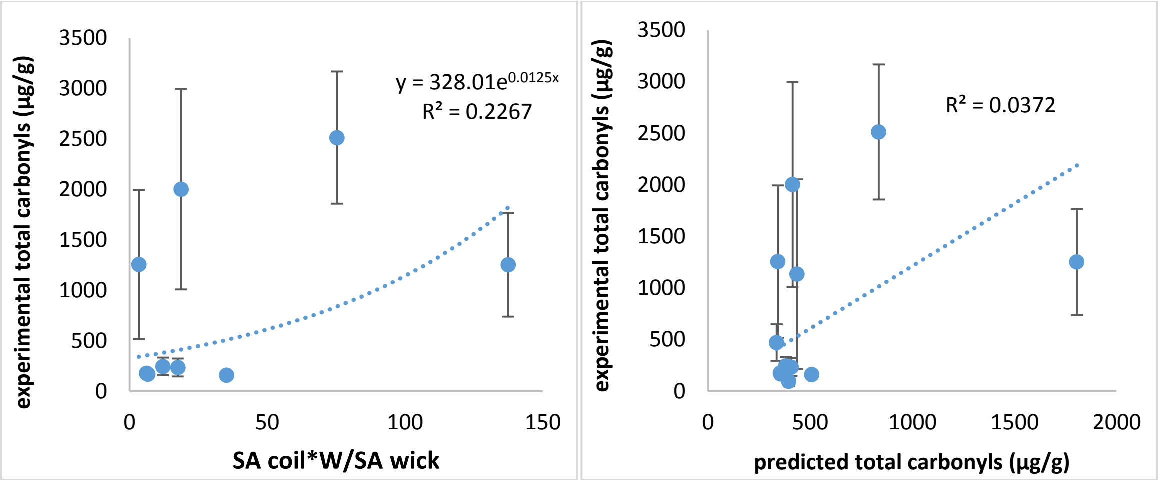

Fig S12. Regression analysis of alternative model (SA coil*Watts/SA wick) (left) and subsequent predictability (right).

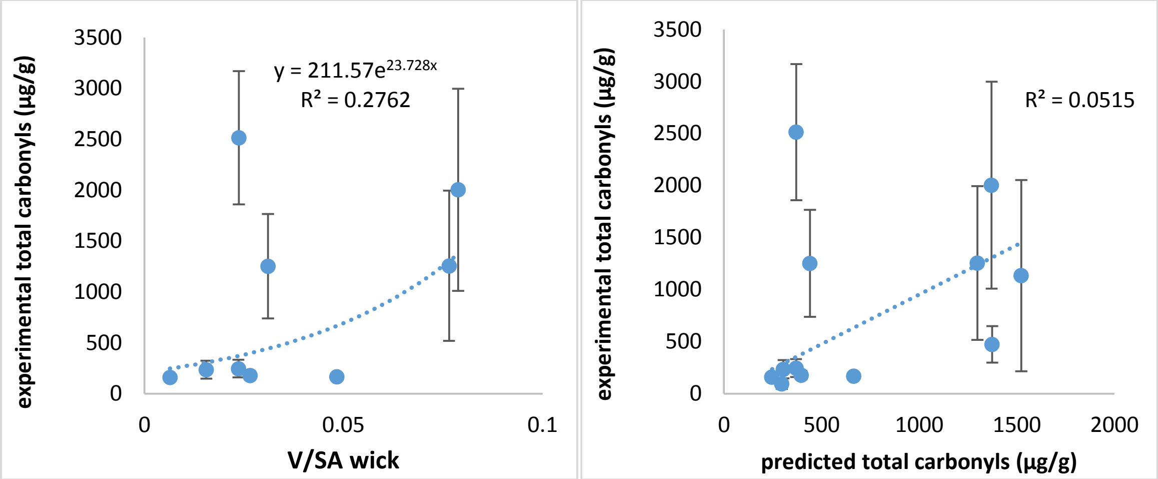

Fig S13. Regression analysis of alternative model (Volts/SA wick) (left) and subsequent predictability (right).

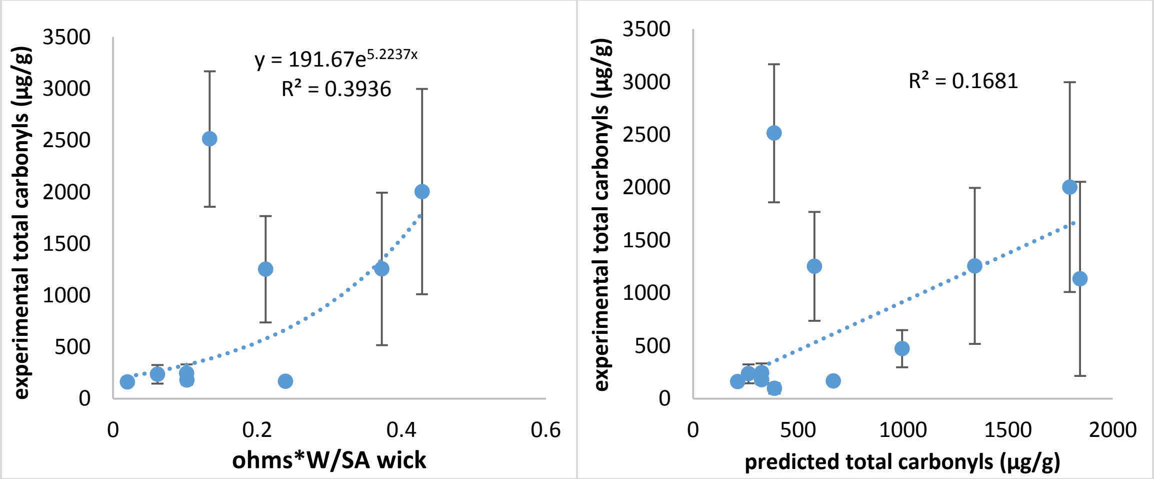

Fig S14. Regression analysis of alternative model (ohms*W/SA wick) (left) and subsequent predictability (right).

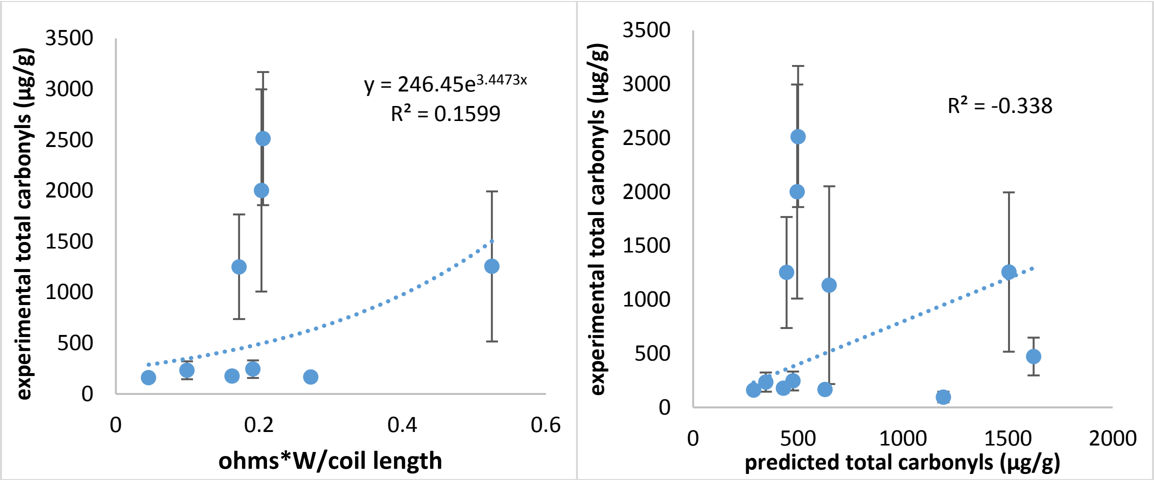

Fig S15. Regression analysis of alternative model (ohms*W/coil length) (left) and subsequent predictability (right).

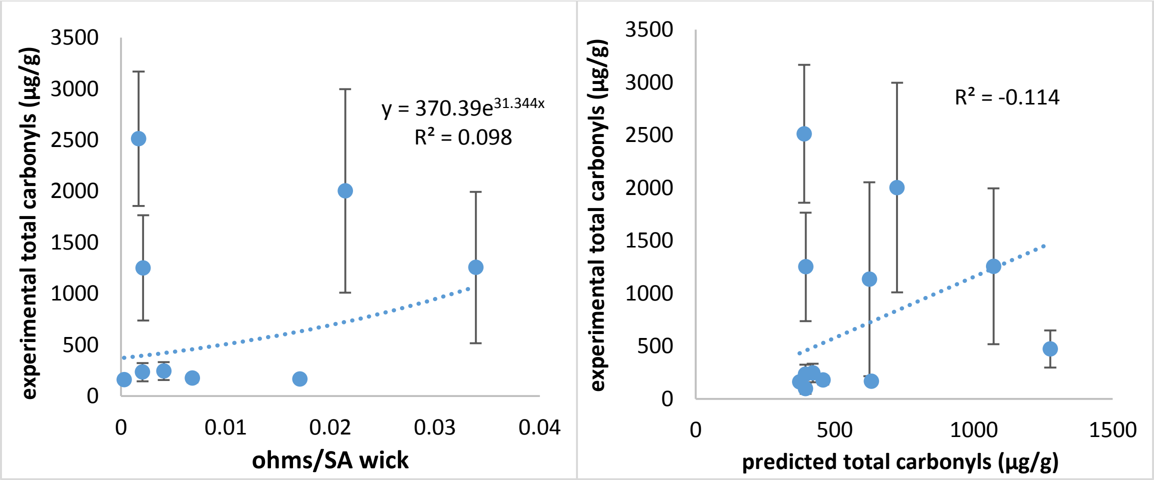

Fig S16. Regression analysis of alternative model (ohms/SA wick) (left) and subsequent predictability (right).
