# Supplementary material for: A simple predictive model for estimating relative e-cigarette toxic carbonyl levels
Source: PLoS One. 2020 Aug 26;15(8):e0238172. doi: 10.1371/journal.pone.0238172 (PMC7449472; doi:10.1371/journal.pone.0238172)
Supplement: S3 Appendix — (PDF) [file pone.0238172.s007.pdf]

Appendix S3.

Additional statistical analysis was performed on the data.

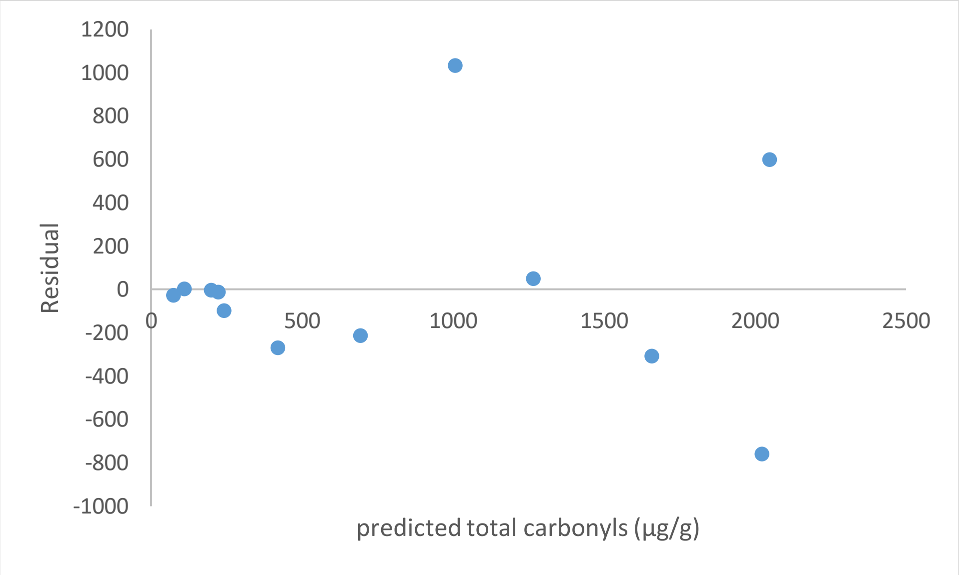

Fig S17. Residual analysis was performed on the relationship between predicted total carbonyl levels and experimental total carbonyl levels of EC1-12 (see Fig. 4). Residuals were plotted against predicted total carbonyl levels and display random dispersion.

Pearson's correlation coefficient was calculated between model 1 and experimental total carbonyl levels of EC1-12 to analyze the association between the two variables. The below Pearson's correlation of .808 identifies a significant association with a p-value of .001.

Table S5. Pearson's correlation coefficient analysis.

		Total Carbonyl Levels
Model 1	Pearson's correlation	.808**
	Sig (2-tailed)	0.001
	N	12

**Correlation is significant (two-tailed)
